# Pore Structure and Deformation Correlation of an Aluminum Foam Sandwich Subject to Three-Point Bending

**DOI:** 10.3390/ma17030567

**Published:** 2024-01-25

**Authors:** Xiaotong Lu, Lei Jing, Wenhao Zhou, Hui Yang, Pingyun Yuan, Xiaocheng Li

**Affiliations:** China Shaanxi Key Laboratory of Biomedical Metal Materials, Northwest Institute for Nonferrous Metal Research, Xi’an 710016, China

**Keywords:** aluminum foam sandwich, melt foaming, hot rolling, pore structure, three-bending test

## Abstract

An Al-Si matrix foam sandwich (AFS) with 6063 Al alloy cover sheets was fabricated by hot rolling combined with melt foaming. A foamable AlSiMg1/SiC_p_ matrix precursor was prepared by the melting route. Hot rolling at 480 °C was carried out to obtain a mechanical bonding interface between the cover sheet and the foamable precursor. Meanwhile, the pore structure of the AFS was deeply affected by the foaming temperature and foaming time during the foaming process. Different pore growth mechanics of the crack-like pore disappearance mechanism (CDM) and pore active expansion mechanism (AEM) were concluded based on the pressure difference in pores inside and outside. Three bending tests were applied to three types of AFSs with different pore structures to evaluate the relation between pore structures and AFS mechanical properties. The bending property of the AFS with fewer layers of pores is like that of a dense material. The bending property of the AFS with a pore size in the range of 0~1 mm presents a typical sandwich shear failure mode. The AFS with a uniform pore structure, in which the shapes of the pores are predominately polygons and the pore diameter is concentrated in the range of 0.5~3 mm, processes a good energy absorption capacity, and the bending stress–strain curve fluctuates greatly after the first stress drop.

## 1. Introduction

An aluminum foam sandwich (AFSs) is a new type of laminar composite that is formed via the bonding of two dense metal cover sheets (Fe, Al, Ti alloy, etc.) and an aluminum foam core [1]. On account of the special sandwich structure, AFSs possess more excellent properties than bare aluminum foam panels [2]. AFSs can be fabricated with complex geometry and adapted to more application areas. Banhart [3] has reported that AFSs can be used with batteries for electric cars, crash absorbers, and high-speed trains as protection against blasts, bullets, and other hazards.

These outstanding properties and a wide range of applications have encouraged the development of various methods for the preparation of AFSs. The essential issues of AFS preparation are interface bonding and the foam core structure. It is known that the properties of materials are determined by their micro- and macro-structures. According to the available studies, the effect of pore structure on foam performance is more obvious than that of microstructures [4]. As to AFS, the factors influencing the performance of AFSs can be extended to the pore structure of the foam core and the interface bonding type between the cover sheet and foam core. The bonding between the metallic cover sheet and foam core can be divided into mechanical and metallic joints [5]. Mechanical bonding can be obtained by adhesives, riveting, screwing, etc. Mechanical bonding can be easily produced at a low cost. But AFSs have additional weight and the joint is low in strength and not resistant to high temperatures. By contrast, the metallic joint AFS with so many advantages (e.g., inflammability, heat resistance, long-term stability, high bonding strength, etc.) can be obtained by rolling [5], soldering [6], hot pressing [7], friction stir welding [8], laser welding [9], and so on. Some research work on fabrication processes for Al foam sandwiches is summarized in Table 1. Hangai et al. [8] employed the friction stir welding route to obtain an AFS that consists of ADC12 Al-Si-Cu die-casting aluminum alloy foam and ADC6 Al-Mg die-casting aluminum alloy face plates. In this process, good bonding between the foam core and cover sheets was formed through friction stir welding. The foam core needs to bond during the foaming process, so the pore structure in the center of the foam core is difficult to control. This may make the center of the AFS an area of weak mechanical properties. It was concluded that a good pore structure and sound ADC6 face plates rely on a suitable foaming time. Compared to using a dedicated device, Neugebauer et al. [10] realized a metallic joint between steel cover sheets and a closed-cell Al foam core by a direct foaming process. The work analyzed the bonding strength of different AFSs (e.g., different foam matrices and various steel types matching each other, metallic bonding, mechanical bonding with glue) using a peeling test. The results show that the glued aluminum foam composites were less resistant in comparison to metallically bonded sandwiches. The above work led to a consensus that the interface bonding force should be higher than the strength of the Al foam core in AFS applications. In addition to the good interface bonding, the foam core structure determines another aspect of the performance of AFSs. Mu et al. [11] conducted quasi-static compression tests on closed-cell Al foam and discussed the deformation mechanisms at the cellular level. Their work revealed the effects of cell shape and foam structure inhomogeneity on compressive deformation. Four failure modes at the cell/membrane level are summarized to understand the energy absorption mechanism of Al foam compression. It is widely accepted that, whether by melt foaming or foaming by the melting of a powder-metallurgically prepared precursor (the precursor means the intermediate products with foaming capacity prepared by the powder or melt method), the cell structure of foam is primarily determined by the foaming parameters, and the number of cell eccentricities (which indicate cell shape) and cell size at the peak of normal distribution determine most of the properties of the metal foam. Kenesei [12] researched the influence of cell-size distribution on the compressive plastic deformation in Alporas foams based on a simple model using Gibson–Ashby cubic arrangements. After analytical and numerical descriptions of the energy absorption capacity as a function of cell-size distribution, it was emphasized that, although the energy absorption capacity is not very sensitive to the cell-size distribution, it is necessary to consider the cell-size distribution in analyzing the compressive energy absorption capacity of metal foam.

The present study aims to fabricate the Al foam sandwich with 6063 Al alloy cover sheets by hot rolling. Understanding the cell structure of the foam core formation process with different foaming parameters and obtaining good bonding between the coversheet and the foam core is key to the preparation and study of AFSs. In addition, the AFS samples were subjected to a three-point bending test, and the effects of cell structure on the AFS bending property were discussed.

## 2. Materials and Methods

### 2.1. Materials

**Figure 1:**
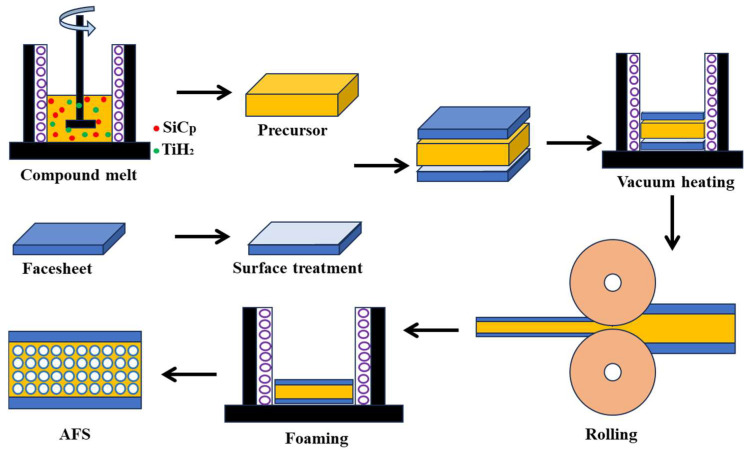
Schematic illustration of the aluminum foam sandwich (AFS) fabrication process.

The fabrication process of AFS in this work can be divided into three steps, precursor—foamable sandwich—AFS, i.e., (1) The foamable precursor is fabricated by the liquid method. The AlSi12 alloy ingot is the raw material prepared by melting to fabricate the core precursor and the foaming agent is TiH_2_. A mixture of 3 wt.% SiC_p_ (~80 μm) is added as a stabilizer to adjust the cell structure. A mixture of 1.5 wt.% Mg is added as an alloying element to enhance the wettability between SiC_p_ (SiC particle) and AlSi12 matrix. (2) The foamable sandwich is provided by hot rolling. The core precursor stacking with 6063 Al alloy cover sheets was sent to the vacuum furnace at 480 °C for 3 h to preheat and then hot-rolled to obtain the foamable sandwich. (3) Rapid foaming was used to prepare the AFS with metallic bonding. The detailed description of the fabrication process was noted in our previous work [5], and the schematic illustration of the AFS fabrication process is shown in Figure 1. The specific process parameters are shown in Table 2, and three sets of specimens were foamed under each condition.

### 2.2. Characterization

The density of the AFS was calculated from the mass-to-volume ratio. The samples’ microstructure was characterized by scanning electron microscopy (SEM; Ultra Plus, Zeiss, Jena, Germany) equipped with an energy dispersive spectrometer (EDS; X-Max, Oxford Instruments, Abingdon, UK), and the results were obtained at an acceleration voltage of 20 kV. The phase structure was analyzed by an X-ray diffractometer (XRD; X Pert Pro, PANalytical B.V., Almelo, The Netherlands) with Cu Kα (λ = 0.154056 nm) radiation. The bending process was recorded by a digital camera (Canon; EOS-1D X MarkII). 

### 2.3. Three-Point Bending Test

**Figure 2:**
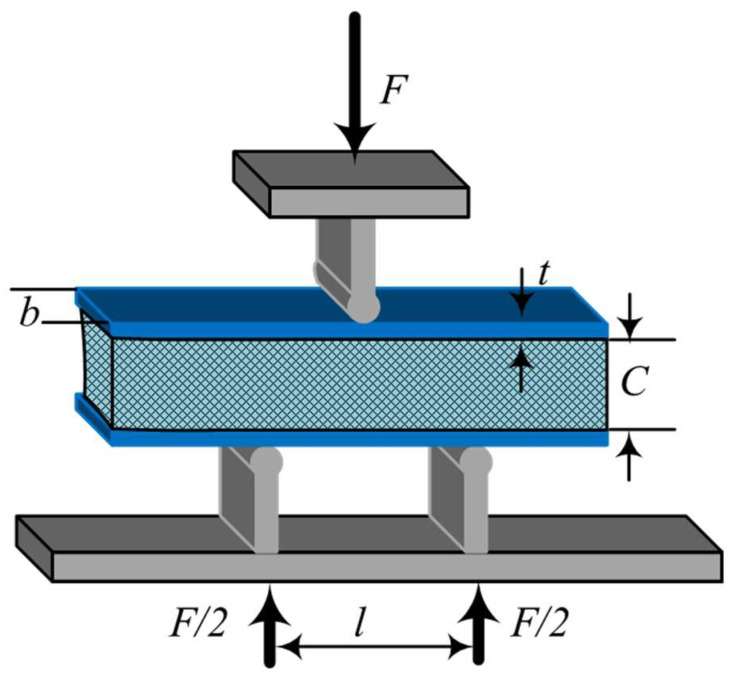
Schematic illustration of the aluminum foam sandwich (AFS) three-point bending test.

Three-point bending tests of the AFS were performed by a universal test machine (WDW-50G, Jinan yongjing Co., Ltd., Jinan, China). Cuboid AFS samples with a size of 50 mm in length, 10 mm in width, and 10 mm in height were cut from AFS panels using electrical discharge machining (ST-2000A, Luoyang Xincheng Precision Machinery Co. Ltd., Luoyang, China). Figure 2 shows the schematic diagram of the AFS three-point bending test. In this work, the strain rate is 10^−3^ s^−1^ at room temperature and the span of bending (*l*) is 20 mm. *C* is the foam core height and *t* is the thickness of the cover sheet. In the present work, the bending behavior of the AFS was described by several indexes: the ultimate failure load (*F_cr_*), bending strength (*σ_b_*), energy absorption capacity (*E_t_*), and specific energy absorption (*E_s_*) were used to evaluate the bending behavior of the AFS. The *σ_b_*, *E_t_*, and *E_s_* are calculated according to the following equations [30]:

(1) $$\sigma_{b}=\frac{3Fl}{2b{\left(2t+C\right)}^{2}}$$

(2) $$E_{t}=\int_{0}^{S_{\max}}Fds$$

(3) $$E_{S}=E_{t}/m_{t}$$

where *m_t_* is the total mass of the AFS.

## 3. Results and Discussion

### 3.1. Microstructure and Morphology

**Figure 3:**
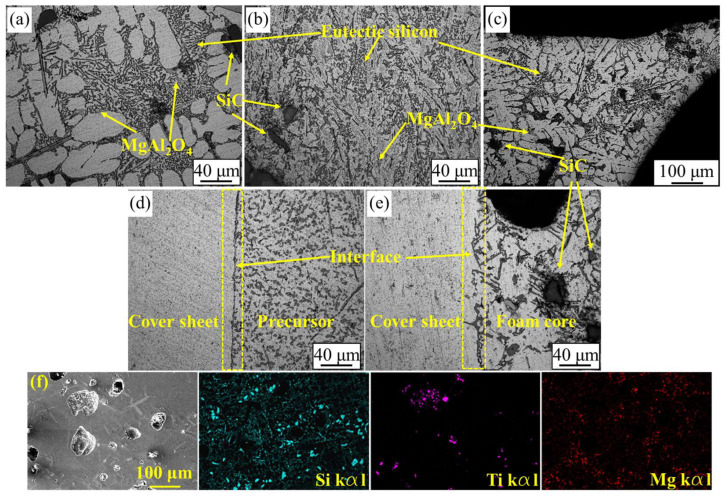
Microstructure of (**a**) the precursor before rolling, (**b**) the precursor after rolling, (**c**) the cell edge of the AFS, (**d**) the interface after rolling, (**e**) the interface after foaming and (**f**) the EDS map of the precursor.

**Figure 4:**
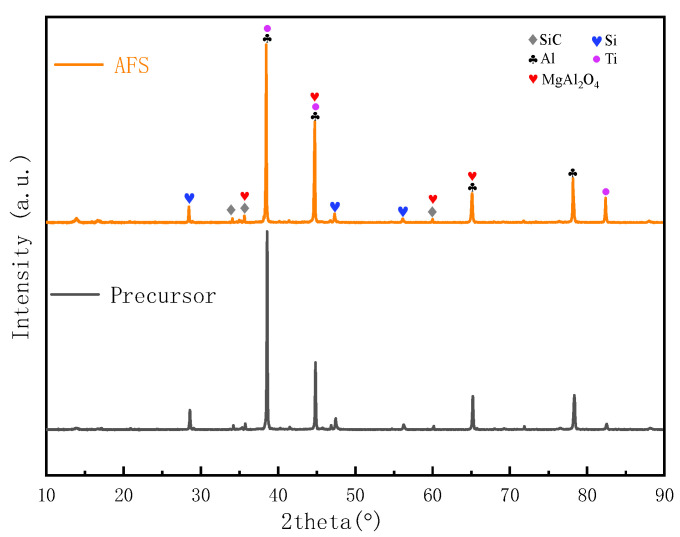
XRD spectra of the precursor and AFS.

To obtain a precursor with good foamability, rapid cooling by water was applied after the addition of TiH_2_ to minimize the release of hydrogen. Meanwhile, the microstructure of the precursor was affected by the fabrication. Figure 3a shows the microstructure of the precursor, in which the bulky dendritic aluminum was surrounded by eutectic Si. This morphology was caused by typical non-equilibrium rapid solidification during water cooling. The microstructure of the precursor after hot rolling is illustrated in Figure 3b; the number of dendritic aluminum grains was decreased and the eutectic Si grains were elongated along the direction of rolling. As to the foaming process, water cooling was also used to obtain the fine pore structure. Thus, the microstructural morphology of each AFS cell edge was similar to that of its precursor before hot rolling. The dendritic aluminum surrounded by eutectic Si became the dominant morphology (Figure 3c). Combined with the XRD spectra of the precursor and AFS (Figure 4), a high temperature refractory spinel phase (MgAl_2_O_4_) was formed in this alloy system. The presence of MgAl_2_O_4_ in the foaming process can restrain the gravity drainage of the melt and stabilize the cell structure [5,31,32,33]. In addition, the SiC particles embedded in the matrix can be seen in all stages of fabrication, and this greatly improves the stability of the foaming process and the uniformity of the cell structure. Figure 3d,e show the interface morphology between the cover sheet and foam core before and after foaming. It is necessary to balance the demand for the good foamability of the precursor with fine interface bonding in hot rolling to ensure the successful preparation of AFS panels [16]. Rolling at low temperatures makes it difficult to obtain good joints and TiH_2_ can decompose to a significant extent at high rolling temperatures. According to the Al-Si alloy phase diagram and the law of TiH_2_ decomposition, the rolling temperature of 480 °C was chosen in the present study. Clear boundaries are found in the interface microstructure after rolling (Figure 3d). This means the interface is mainly mechanically bonded. The AFS core matrix remelts and solidifies in the foaming process, which can facilitate the joint between the cover sheet and foam core changing from mechanical bonding to metallic bonding. As shown in Figure 3e, no boundaries can be seen in the interface after foaming, the cover sheet and foam core layer are integrated and metallic bonding is achieved. In the precursor fabrication, a little TiH_2_ decomposition seems inevitable under rapid cooling, with some micro-pores nucleating and growing in the precursor. Figure 3f shows the EDS analysis of the precursor. Some micro-pores with a diameter of about 10–200 μm are embedded in the matrix, the Si and Mg elements are uniformly distributed on the matrix, and Ti mostly gathers in the bottom of each cell. The XRD result shows that the characteristic peak intensity of Ti in the AFS is stronger than that in the precursor. It may be caused by the decomposition of TiH_2_; the face-centered cubic TiH_2_ was transformed to body-centered cubic Ti during the foaming process [34].

### 3.2. Foaming Process

**Figure 5:**
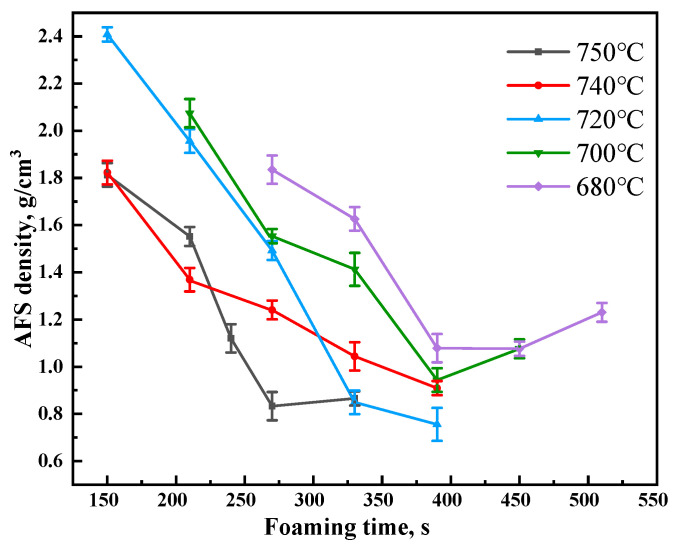
Variation in the AFS density at different foaming times and temperatures.

The foaming temperature and foaming time are key parameters of the foaming process [35]. The proper foaming parameters not only provide the driving force for the decomposition of TiH_2_, but also have an important influence on the melt viscosity and the growth of pores. After hot-rolling, the density of the foamable sandwich changed from 1.9 g/cm^3^ to 2.59 g/cm^3^. The Al-Si binary phase diagram indicates that the melting point of the AlSi_12_ alloy is 577 °C. To balance the melting rate of the matrix and rate of decomposition of TiH_2_, the foaming temperatures range from 680 °C to 750 °C. Figure 5 depicts the variation in the AFS density at different foaming times and temperatures. The variation in the AFS density at different temperatures is similar to that with changing foaming time. All the samples experienced pore nucleation, pore growth, pore coalescence, and pore collapse. In the first two stages, the AFS density rapidly decreases with foaming time, then the density slowly decreases when it encounters pore coalescence. As to the stage of pore collapse, the AFS density increases instead: this is because the pore collapse stage belongs to the late stage of foaming, the gas in the melt at high temperatures will be discharged over time, so that the total amount of gas involved in the formation of pores will be reduced; on the other hand, with the prolongation of the foaming time, the pore collapse leads to an increase in the proportion of the solid aluminum layer in the core layer of the AFS while the proportion of the foam layer decreases, so that in this stage of the preparation process, the AFS porosity will be reduced, and the density will be increased [36,37]. 

#### 3.2.1. Effect of Foaming Temperature on Pore Structure

**Figure 6:**
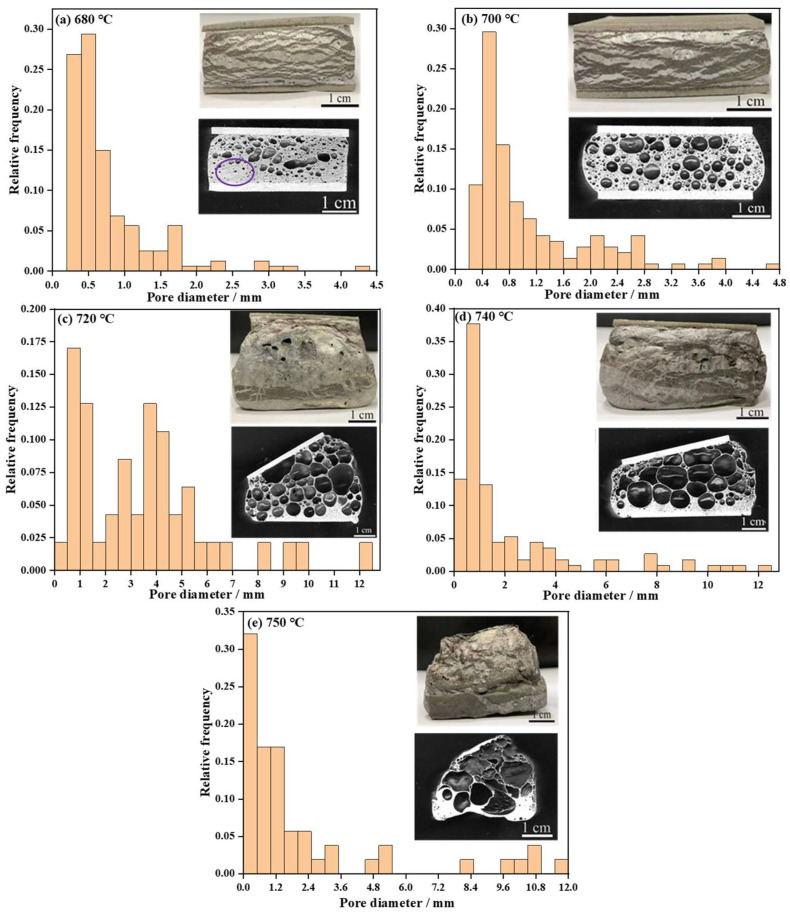
Pore structure image and histogram of diameter distribution at different temperatures for 330 s (**a**) 680 °C, (**b**) 700 °C, (**c**) 720 °C, (**d**) 740 °C, (**e**) 750 °C.

To evaluate the effect of temperature changes on AFS foaming behavior, the evolutions of the pore structure in terms of their diameter distributions as a function of temperature from 680 °C to 750 °C are shown in Figure 6 (where a constant foaming time of 330 s was used). This shows that the effect of temperature on the pore structure is particularly significant. At 680 °C, the low temperature is lacking the driving force of cell growth, so a number of micro-pores with diameters mostly concentrated around 0.5 mm are formed. The pore shapes are dominated by polygonal micro-pores and some long narrow pores (Figure 6a). Most pores become rounded at 700 °C (Figure 6b), and the increase in the pore mean diameter did not result in significant macroscopic expansion of the AFS. At both 680 and 700 °C, the AFS had not completely expanded. At 720 °C, the maximum expansion of the AFS was achieved and the pore size increased significantly. The pore sizes were mostly distributed within the range of 2–6 mm, the diameter of a few pores even exceeded 10 mm with sufficient foaming. To explore the foaming behavior of the AFS in this study, no measures have been taken to adjust the horizontal position of the cover sheet in foaming process. The distinct incline of the cover sheet caused by expansion of the foam core can be seen in Figure 6c. Compared with Figure 6c, the pore size increases but the height of the AFS decreases. The differences in pore morphology and size are caused by pore coalescence at this stage. The result corresponds to Figure 5, which shows that the AFS density at 720 °C is higher than that at 740 °C. When the foaming temperature reaches 750 °C, a big cavity occupies most of the area of the foamed core and the cover sheet disappears due to the high temperature. Based on these results, when the foaming time is 330 s, the optimum foaming temperature of the AFS is 720 °C. At lower temperatures, some serious problems are encountered, such as the worst expansion rate of the AFS (it can be concluded in Figure 5 and can also be obtained by comparing the AFS morphology images with the different foaming temperature in Figure 6). The pore structure is non-uniform (the morphology shown in Figure 6a), and the foam core does not achieve complete expansion (see the purple frame labeling in Figure 6a), etc. The foaming mold collects heat to a lesser extent at a low temperature than that at a high temperature, and the heating rate of the precursor at low temperature is so slow that it leads to severe oxidation of the precursor. The oxides on the surface of the precursor can significantly impede the foaming process. Meanwhile, it is difficult to balance the optimal foaming temperature of the precursor with the decomposition temperature of TiH_2_ at a low temperature, which reduces the utilization ratio of TiH_2_, thereby impeding pore growth [38]. When the foaming temperature is too high, local overheating of the foam core can occur in the foaming process. The high temperature of any precursors will lead to the premature bursting and merging of the formed bubbles, and eventually lead to the collapse of, or a big cavity in, the foam core. The selection of foaming temperature is crucial to obtain an AFS with a good pore structure.

#### 3.2.2. Effect of Foaming Time on Pore Structure

**Figure 7:**
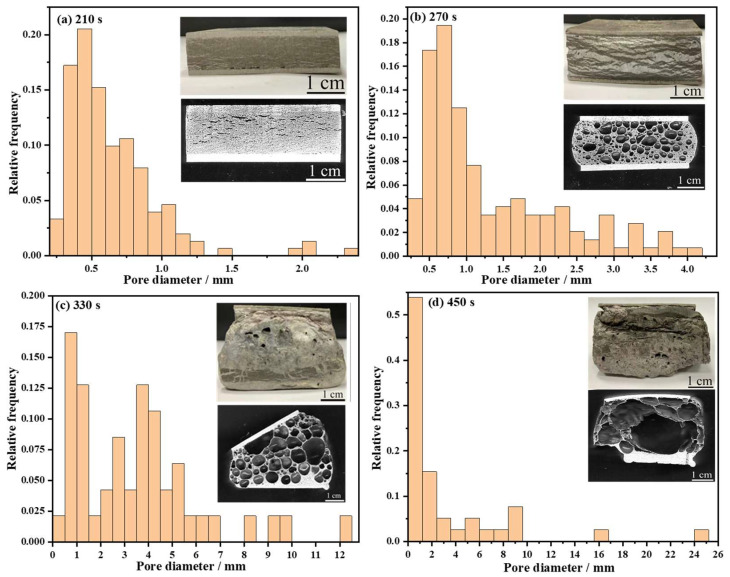
Pore structure image and histogram of diameter distribution with different foaming times at 720 °C (**a**) 210 s, (**b**) 270 s, (**c**) 330 s, (**d**) 450 s.

Figure 7a–d show the pore structure image and histogram of the diameter distribution of the AFS with different foaming times at 720 °C, revealing the evolution of the AFS morphology and pore size over time. Many crack-like pores can be seen in Figure 7a and the AFS has barely expanded upon foaming for 210 s. The pores are difficult to grow within such a short foaming time and the pore sizes are concentrated in the range of 0.25~0.75 mm. There are micro-pores and polygonal holes in the foam core and the crack-like pores disappear when the foaming time is extended to 270 s. The pore sizes are mainly distributed in the range of 0.5~1.5 mm (Figure 7b). As shown in Figure 7c,d, the pores in the foam core stabilize by 330 s, then merge to form a large cavity at 450 s.

#### 3.2.3. Foaming Mechanism of AFS

**Figure 8:**
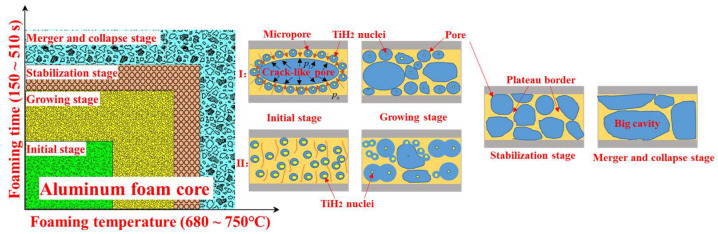
Schematic illustration of two foaming mechanism AFSs.

To understand the foaming process of AFS, two foaming mechanisms are deduced (Figure 8). The foaming process can be divided into four stages with the variation in foaming temperature and time, i.e., initial stage, growth stage, stabilization stage, followed by a merger and collapse stage. The foaming mechanism of AFS can be seen to be a crack-like pore disappearance mechanism (CDM) and active expansion mechanism (AEM). It can be seen from Figure 6a that in the initial stage of foaming, there are many crack-like pores which lie perpendicular to the direction of rolling in the foam core and these are surrounded by some micro-pores. The phenomenon also arises in precursors fabricated by powder metallurgy routes [39]. A simplified form of the Rayleigh equation is introduced to support the CDM model, and the Rayleigh equation for a gas–liquid interface during the foaming process of a single bubble in a metallic melt is given by [40]:

(4) $$p_{pore}=p_{c}+\rho gh+\frac{2\sigma }{R}$$

where *p_pore_* is the pressure of a single pore, *p_c_* denotes standard atmospheric pressure, *ρ* is the density of the metallic melt, *g* represents the acceleration due to gravity, *h* is the pore depth located in the melt, *R* is the radius of curvature of a pore in the metallic melt, and *σ* is the surface energy of the metallic melt (in this study, *σ* is a constant). The pressure difference (∆*p*) between the pressure of the crack-like pore (*p_i_*) and the total pressure of micro-pores (*p_o_*), so ∆*p* can be calculated using Equation (5):

(5) $$\Delta p=p_{i}-p_{o}=2\sigma (\frac{1}{R}-\frac{n}{\bar{r}})$$

where $\bar{r}$ and *n* are the average radius and the number of the micro-pores around crack-like pores, respectively. Based on the work of Wang et al. [38], ∆*p* during the initial stages of foaming is quite large and decreases quickly with increased foaming time, suggesting that the crack-like pore disappears and the difference ∆*p* is generated mainly in the initial stage of foaming and the crack-like pores vanish completely during the growth stage. The H_2_ is also decomposed by TiH_2_ in the growing stage, prompting the expansion of the pores. Adjacent bubbles are approximately the same size as the growing pores; according to Equation (5), the ∆*p* values in adjacent bubbles are quite small, or even close to zero. In the meantime, the stabilization stage of the foaming process takes place. The stabilization stage is very short; the corresponding temperature and time are the optimal foaming parameters [41]. In time, and with increasing temperature, the TiH_2_ decomposes completely and the pores begin to merge and collapse into large cavities (Figure 6d,e). The change in the pores is dominated by drainage under gravity and capillary action [2]. The AEM model illustrates the foaming mechanics of the core without crack-like pores in the initial stage. In the initial stage, the solid particles in the foam core can increase the nucleation rate of pores. The pores were distended because of their being filled by hydrogen, but the growth was restricted by gravity drainage under molten conditions and the surface tension of the melt [1]. Due to the shrinking core mechanics driving the TiH_2_ decomposition process, the rate of expansion of H_2_ decreases in time [42], so pores grow rapidly in the initial stage by the decomposition of TiH_2_. As the pores nucleate and grow rapidly, the foaming process reaches the growth stage and the rate of release of H_2_ decreases. In this stage, under the action of surface energy, the big pores swallow the small pores to obtain the optimum rate of AFS expansion. The stabilization stage and merger and collapse stage of AEM are similar to those of CDM.

### 3.3. Three-Bending Test

To explore the relationship between the pore structure and mechanical properties of the AFS, three AFS samples with different pore structures were selected for the three-point bending test. According to Li et al. [43] and Zu et al. [30], the failure modes of AFS subjected to three-point bending include indentation, core shear, and face yield. The failure modes are affected by several factors [40], such as the materials used for the cover sheet and foam core, the pore structure of the foam core, the density of the AFS, etc.

#### 3.3.1. Pore Structure of AFS Samples

**Figure 9:**
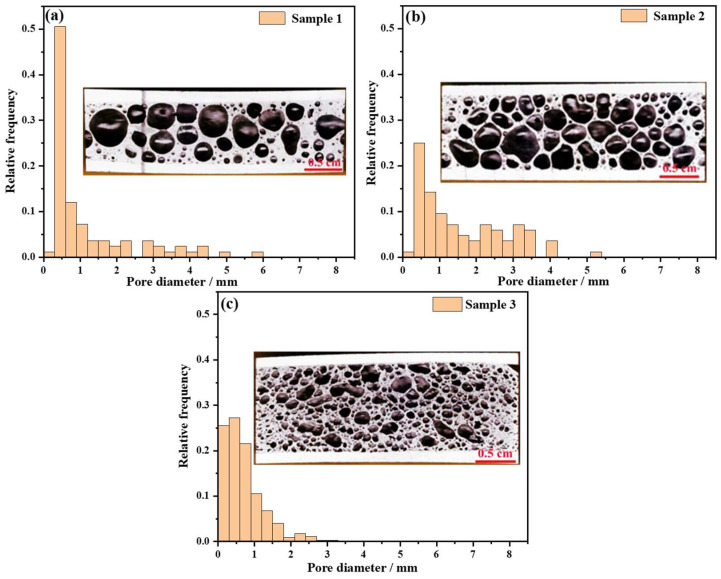
Pore structure image and diameter distribution histogram of bending samples: (**a**) Sample 1, (**b**) Sample 2, (**c**) Sample 3.

Figure 9 shows the pore diameter distribution and the number of pores in different diameter ranges. The macro-morphology demonstrates that the pore diameter of Sample 1 is polarized. In Figure 9a, more than 60% of the pores are less than 1 mm in diameter, and these micro-pores are formed by the flow of melt towards the junction of the plateau border. In addition, there are some pores larger than 3 mm in diameter and the shape of these pores are mostly round, which governs the mechanical properties of the aluminum foam. The uniform pore structure is illustrated in Figure 9b, and the shapes of these pores are dominated by polygons. The pore diameters are concentrated within the range of 0.5~3 mm and the proportion of micro-pores is significantly reduced. The pore diameter of Sample 3 is mostly within 1.5 mm and the number of pores is increased. The cell wall thickness decreases from Sample 1 to Sample 3.

Table 3 summarizes the effect of the pore structure of the foam core on the bending strength of the AFS. In this experiment, the density of Samples 1, 2, and 3 is 1.352 g/cm^3^, 1.023 g/cm^3^, and 1.201 g/cm^3^, respectively. The difference in the AFS density is mainly attributed to the pore structure. As shown in Figure 9, the percentage of cell wall in Sample 1 is higher than that in Samples 2 and 3, and the cell wall and pore structure of Sample 2 are more uniform than those of Samples 1 and 3. Combined with Table 3, Sample 1 has the highest bending strength and elastic modulus among the three samples, reaching 49.9 MPa and 857.46 MPa, respectively. The energy absorption capacity is calculated from the stress–strain curve and the strain ranges from 0 to 0.5. The results show that Sample 2 possesses the maximum energy absorption capacity in flexure.

#### 3.3.2. Three-Bending Deformation Process

**Figure 10:**
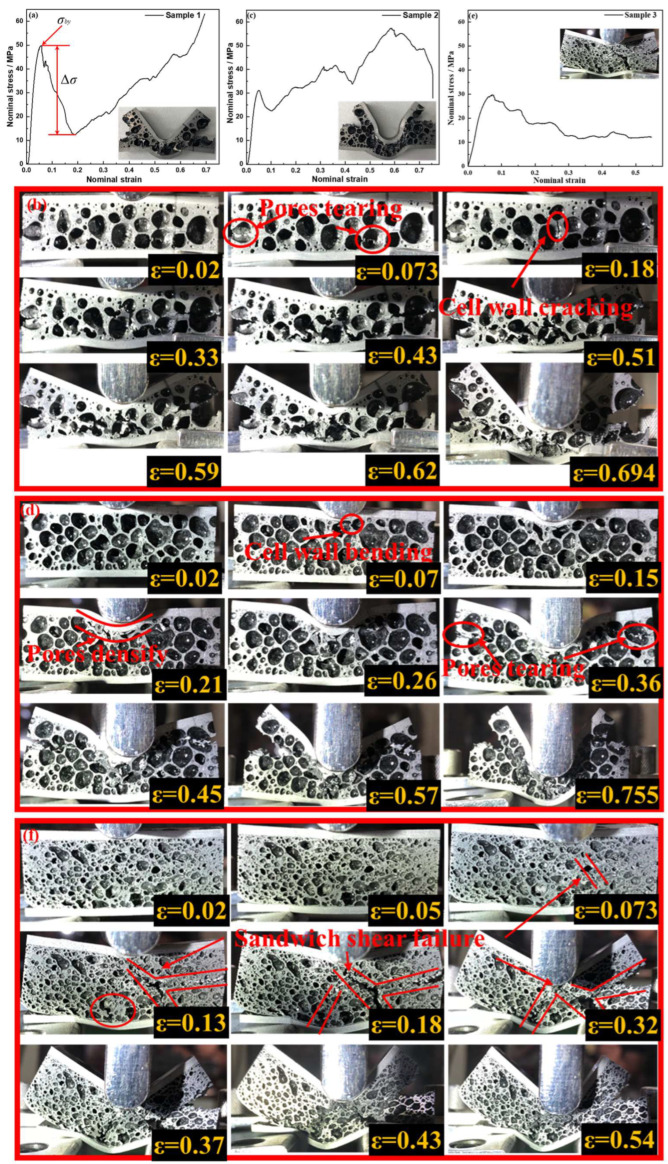
Stress–strain curve of AFS under three-bending test (**a**) Sample 1, (**c**) Sample 2, and (**e**) Sample 3; bending deformation process of (**b**) Sample 1, (**d**) Sample 2, and (**f**) Sample 3.

The AFS samples with different pore structures have different bending behaviors, so the mechanism of AFS bending can be understood by way of the deformation thereof. Figure 10 shows the three-point bending process of the AFS samples; the stress–strain curves display various tendencies due to the discrepancies in the pore structures across the three specimens. It can be found that the stress–strain curve exhibits three distinct regions: a linear elastic region at low strain; a rapid stress drop (∆*σ*) region once the load exceeds the yield point; and a loop densification region with stress fluctuation over a wide range of the strain. Figure 10a demonstrates the bending stress–strain curve of Sample 1, in which the stress drop reaches to 36.5 MPa. Next, a sharp drop in the curve can be seen after a short rise, then the stress increases continuously with the strain. Combined with Figure 9a, the greatest number of micro-pores can be found in the thick wall and a few pores with large diameter are found in the foam core (Sample 1). The typical characteristic leads to the high *σ_by_* and large stress drop; because of the few pore layers, pore collapse occurred in the first two stages and the densification stage began immediately, so the bending stress–strain curve keeps rising after the first stress drop. Figure 10b shows the bending deformation process of Sample 1, in which sample damage begins with the pore-bottom tearing and cell wall cracking (marked with a red circle); *ε* = 0.073 in Figure 10b corresponds to the end of the elastic phase in Figure 10a. Then, the pores start to collapse and densify under increasing stress, when the cell-wall cracking is observed in Figure 10b (*ε* = 0.18), and the ∆*σ* region has finished as shown in Figure 10a. At the end, the foam core is completely destroyed and the bonding interface suffers no damage. Under bending stress, the cover sheets bear tensile and compressive stresses and the foam core bears the shear stress [30]. Figure 10c shows the bending stress–strain curve of Sample 2. Compared to Sample 1, the curve fluctuates greatly after the first stress drop, showing an overall upward trend. The pore structure and cell wall thickness of Sample 2 are more uniform than those of Sample 1. From Figure 10d, the pores directly below the indenter are damaged first. Normal stress makes the cell wall bend and rupture (as marked by the red circle), then the collapse of pores occurs. The cell wall bending (*ε* = 0.07) in Figure 10d indicates the end of the elastic phase in Figure 10c. The next pores then bear the load, the curve in Figure 10c begins to rise until the pores densify (*ε* = 0.21) and another small stress drop occurs. The area below the indenter forms a dense layer as the strain increases (marked by the red line). In addition, the foam core is subjected to shear stresses, so the pores on both sides of core are torn and become the starting point of cracking (marked by the red circle). As the strain increases, the crack in the foam core extends and the pores are collapsed layer-by-layer to form a dense area. Figure 10e shows the typical sandwich shear failure stress–strain curve of Sample 3. The AlSi12 alloy foam core is more brittle under bending tests [5]. As illustrated in Figure 10f, the broken area of Sample 3 is marked by the red line which presents “T” and “X” shapes. This indicates that the foam deforms in brittle shear. Differing from Samples 1 and 2, the pore structure of Sample 3 has the most irregularly shaped micro-pores and the proportion of cell wall achieves 41.62%. The irregular micro-pores scatter in the AlSi_12_ matrix as if they are tiny defects, which causes severe stress concentration in flexure. The stress concentration promotes the brittle shearing of the foam core.

#### 3.3.3. Energy Absorption Capacity

**Figure 11:**
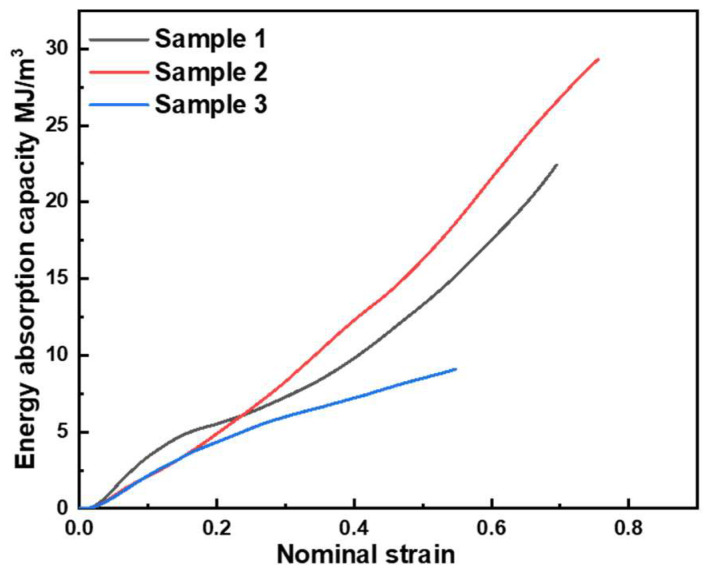
Energy absorption capacities of three samples under the bending test.

The energy absorption capacity of the samples is mainly determined by the deformation in bending. Figure 11 shows the energy absorption capacity of the three samples. Sample 1 has a higher energy absorption capacity within the strain of 0.2 than those of Samples 2 and 3. Table 3 summaries the elastic modulus of the three samples and shows that *E*_1_ > *E*_2_ > *E*_3_. Due to the higher elasticy modulus of Sample 1, the bending strength in the linear elastic region is maximized at 49.9 MPa. However, the non-uniformity of the pore structure and the small number of pore layers can induce a greater stress drop. After the first stress drop, the bending energy is dissipated mainly in the process of pore compaction, bending of cover sheets, and friction between pore walls. The energy absorption capacity of Sample 2 tends to be weaker. Combined with Table 2, Sample 2 has the best energy absorption capacity. Samples 2 and 3 have the same energy absorption capacity in the linear elastic region. Compared to Sample 1, the elastic deformation is found in the cover sheet and AlSi_12_ foam core in the linear elastic region. The bending strengths of Samples 2 and 3 are about 30 MPa. However, in the linear elastic region of Sample 1, the foam core suffered a shear tear at the bottom of the biggest pore before elastic deformation of the cover sheet and AlSi_12_ foam core. As depicted in Figure 10c, Sample 2 has a uniform pore structure and cell wall. Sample 2 exhibits the dissipation of bending energy by pore collapse and compaction. In addition, the large number of pore layers causes the long compaction area. Thus, Sample 2 shows an excellent energy absorption capacity. Figure 10 presents that Sample 3 is the first to be completely destroyed in the test. The shear fracture prematurely appears on the foam core layer, and the structure loses its bearing capacity. As a load-bearing buffering energy-absorbing structure, it is very unfavorable to absorb impact energy and cannot play a good buffering and protection role. The typical structure (Sample 3) has a small yield strain and no obvious plateau stress, giving rise to the poor absorptive capacity.

#### 3.3.4. Fracture Morphology Analysis

**Figure 12:**
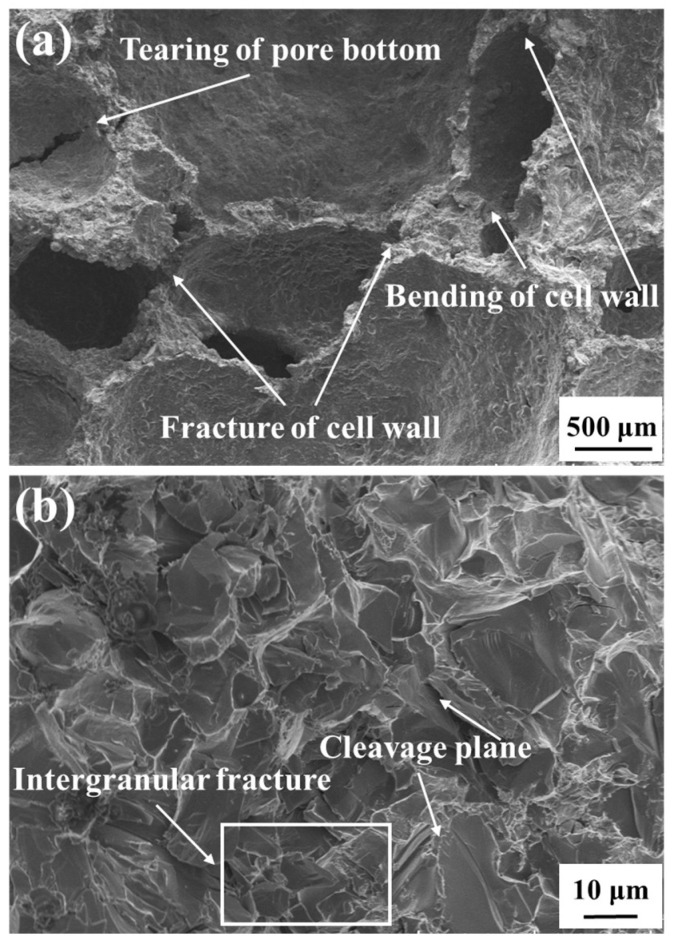
SEM micrographs of the fracture surface of the AFS samples after three-point bending: (**a**) low magnification and (**b**) high magnification.

SEM micrographs of the fracture surface of the AFS samples are shown in Figure 12. The AFS sample deformations (tearing of the pore bottom, bending of the cell wall, and fracture of the cell wall) can be seen in Figure 12a, verifying the role of pore deformation in the bending process. As the AlSi_12_ matrix comprises the brittle Al alloy, the AFS samples are all brittle materials. The transcrystalline brittle fracture surface and cleavage planes in Figure 10b indicate the brittle fracture of the AFS in bending. According to Zu et al. [30,44], in Al-Si alloy matrix foam, the cracks are generated across the a-Al/Si eutectic zone and the brittle eutectic phases. In addition, the bending behavior of the AFS is mainly dependent on the foam core structure and cover sheet; the microstructure of the foam matrix has little effect on the mechanical behavior of the AFS [4,45].

## 4. Conclusions

AFS panels consisting of AlSi_12_ foam and 6063Al alloy cover sheets were fabricated by melt foaming and hot rolling. Compared with other preparation methods, it not only reduces the cost of raw materials, but also reduces the need for machining capacity [46,47,48]. It has great prospects for industrialization and application. The metallic bonding between the foam core and cover sheet was realized in the fabrication process. In addition, the relationship among the foaming process, foaming structure, and bending properties was explored. The main conclusions can be summarized as follows:

- The foamable precursor was prepared by the melting route. Rapid cooling followed immediately after the addition of TiH_2_ to minimize the release thereof;
- In the hot-rolling process, vacuum heating to 480 °C was applied over 3 h to prevent interface oxidation and obtain good bonding. The interface was mainly fixed by mechanical bonding with a little metallic bonding in some areas after hot rolling;
- In the foaming process, two kinds of foaming mechanism were concluded, i.e., a crack-like CDM and pore AEM. These two foaming mechanisms are all based on the pressure difference between the inside and outside of the pores. In addition, the metallic bonding of the interface was obtained after this foaming process. The microstructure of the foam matrix, if changed by hot-rolling, can be recovered in the foaming process;
- The pore structure and pore size affect the bending properties of the AFS. Fewer pore layers in the AFS result in a significant stress drop and then the bending properties become more akin to those of a denser material. However, the pore diameter of the AFS distributed mainly in the range of 0~1 mm presents a typical sandwich shear failure mode. The AFS with a uniform pore structure has a polygonal pore shape and the pore diameters are concentrated in the range of 0.5~3 mm, giving rise to a good energy absorption capacity, and the bending stress–strain curve fluctuates greatly after the first stress drop;
- The SEM images of the AFS bending fracture morphology show multiple brittle fracture characteristics in such samples, proving that the AlSi_12_ matrix foam core fails in brittle fracture under bending.

## Figures and Tables

**Table 1:** Some research works of fabrication processes for Al foam sandwich.

Bonding Type	Fabrication Method	Foam Core Structure	Cover Sheet	References
Metallic joints	Extrusion	C-AlSi7 foam	AlMn1	[13]
	Hot pressing	C-AlMg4Si8 foam	Al	[14]
	Direct foaming to bond coversheet	C-AlSi10 foam	Stainless steel wire mesh-grid	[15]
	Friction stir welding	C-ADC12 foam	ADC6 plate	[8]
	Hot pressing	C-AlSi12 foam	Q235 steel	[16]
	Self-propagating high-temperature synthesis	O-Al foam	Al	[17]
	Direct foaming to bond coversheet	C-Al foam	Steel sheet	[10]
	Laser welding	O-AC2A foam	A6063	[9]
	Soldering	O-Al foam	316 Steel sheet	[18]
	Hot rolling	C-AlSi12 foam	A6063	[19]
	Hot rolling	C-AlSi7 foam	Al	[20]
	Soldering	C-Al foam	5056 Al	[6]
Mechanical joints	Adhesive bonding	Alporas foam	Stainless steel	[21]
	Adhesive bonding	C-7075Al foam	Carbon fiber	[22]
	Adhesive bonding	C-Al foam	Al-1060	[23]
	Adhesive bonding	C-Al foam	5052 Al	[24]
	No joining	C-Al foam	Steel sheet	[25]
	Inorganic adhesive bonding	C-Al foam	Al (99.2%)	[26]
	In injection process	O-A356 alloy foam	Carbon fabric	[27]
	Adhesive bonding	C-Al foam	Al (99.5%)	[13]
	Adhesive bonding	Alporas foam	AA 1100-O	[28]
	Adhesive bonding	Alporas foam	AA 1100-O/AA 3104-H19	[29]

Where C- and O- denotes closed-cell and open-cell, respectively.

**Table 2:** Preparation process parameters of the AFS.

	Temperature (°C)	680	700	720	740	750
Time (s)						
150		✕	✕	√	√	√
210		✕	√	√	√	√
240		✕	✕	✕	✕	√
270		√	√	√	√	√
330		√	√	√	√	√
390		√	√	√	√	✕
450		√	√	✕	✕	✕
510		√	✕	✕	✕	✕

Where √ and ✕ represent experiments performed and not performed, respectively.

**Table 3:** Bending test results of the AFS samples.

	Density (g/cm^3^)	Bending Strength (*σ_by_* MPa)	Energy Absorption Capacity (*E_t_* MJ/m^3^)	Specific Energy Absorption (*E_s_* MJ/m^3^g^−1^)	Elasticity Modulus (*E*)	Foaming Temp. (°C)	Foaming Time (s)
Sample 1	1.352	49.9	13.3	1.42	857.46	700	330
Sample 2	1.023	31.3	16.3	1.87	637.91	720	270
Sample 3	1.201	29.6	8.5	0.79	484.33	680	450

where *σ_by_* is the bending strength at yield point.

## Data Availability

The original contributions presented in the study are included in the article, and further inquiries can be directed to the corresponding authors.
